# Croatian national audit on videolaryngoscopes and alternative intubation devices in the era of COVID-19 pandemic

**DOI:** 10.1371/journal.pone.0280236

**Published:** 2023-01-11

**Authors:** Marijana Matas, Martina Miklić Bublić, Ante Sekulić, Renata Curić Radivojević, Bálint Nagy

**Affiliations:** 1 Department of Anesthesiology, Reanimatology and Intensive Care Medicine, University Hospital Center Zagreb, Zagreb, Croatia; 2 School of Medicine, Catholic University of Croatia, Zagreb, Croatia; 3 Department of Anesthesiology and Intensive Therapy, Medical School, University of Pécs, Pécs, Hungary; 4 Medical Skills Lab, Medical School, University of Pécs, Pécs, Hungary; Sapienza University of Rome: Universita degli Studi di Roma La Sapienza, ITALY

## Abstract

**Introduction:**

Videolaryngoscopy (VL) is the recommended strategy for airway management in COVID-19 patients and guidelines recommends that all anesthesiologists should be trained to use and have immediate access to the device. However, the availability of VL in hospitals and its use may vary, as well as the choice of the device and necessary training. Our primary aim was to investigate data on availability of VL in Croatia, its use, the choice of the device and its implementation, with special consideration of COVID-19 management.

**Materials and methods:**

An electronic survey was sent to all Croatian hospitals that have anesthesiology service available. The survey was designed to examine data on availability and use of VL with special consideration of COVID-19 wards. The survey was conducted between 1.03.2021 and 30.08.2021.

**Results:**

Response rate was 83%. VL was available in 86% of hospitals and the best supplied areas were intensive care units, general surgery and gynecology/obstetrics. The most common VL devices were Bonfils, C-MAC and C-MAC D-blade. The choice of VL was mainly based on centralized hospital procurement and informal introduction was found to be the most frequent training method. The VL was mainly used in Croatian hospitals in cases of difficult airway or as a backup method after failed intubation. Only 16% of hospitals reported regular use in everyday practice. Even though, VL was available in 64% of COVID-19 wards, only 21% of hospitals reported routine use.

**Conclusion:**

Although VL is available in the majority of Croatian hospitals, its use is still mainly restricted to difficult airway scenarios. Use of VL in COVID-19 management is also low and education on the method is still mainly informal. Based upon our results better implementation in practice should be targeted, as well as formal skill trainings especially regarding COVID-19 care.

## Introduction

Based on recent reports, the rate of difficult intubation is 1.6 per 1000 patients, with an estimated incidence of adverse events (AE) up to 1 in 5500 cases, with an increase in emergency department and ICU settings [1,2]. Even though, direct laryngoscopy remains the standard for airway management, development of videolaryngoscopy (VL) represents a significant advance in airway management especially regarding difficult airways. VL offers benefits including superior laryngeal view, reduction in applied force, improved first-attempt success rate, as well as high rescue intubation success rate and shorter learning curve [3–7]. Furthermore, the following additional benefits of VL became recognized recently regarding the COVID-19 pandemic: lower risk of cross-contamination, improved visibility and increased first-attempt success rate in personal protective equipment and lower risk of failed intubation [8–11]. Based on the aforementioned advantages, VL is recommended as first-line strategy for airway management in COVID-19 patients and Difficult Airway Society 2015 guidelines recommends in general that all anesthesiologists should be trained to use VL and have immediate access to the device [12–14].

Despite the previously mentioned recommendations and guidelines, availability of VL in hospitals and its use in airway management may vary, as well as the choice of the device and necessary training [15,16]. To our knowledge, there is no published data so far regarding the availability of VL in Croatian and national data in general are sparsely published in this field as well. Therefore, our primary aim was to investigate data on availability of VL in Croatia, its use, the choice of the device and its implementation, with special consideration of COVID-19 management.

## Materials and methods

**Fig 1:**
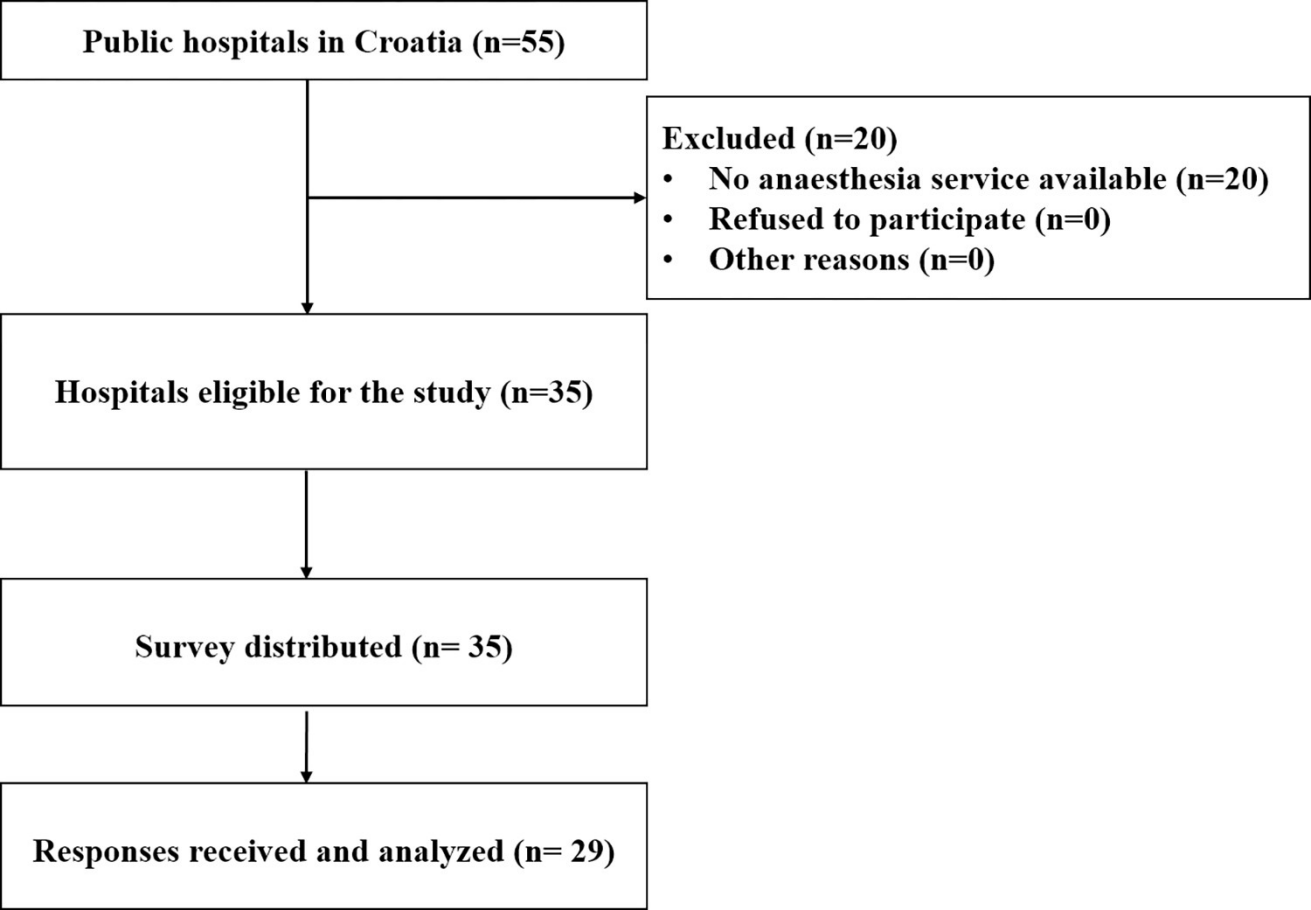
Flow diagram of the study.

The study was designed by the authors and questions regarding availability and usage of VL are shown respectively in Croatian and in English in Supplement 1 and 2. Prior to this study, permission was first obtained from the Institutional Review Board at the University Hospital Centre Zagreb, Zagreb, Croatia (registration number: 8.1-21/17-2). The study was conducted between 1.03.2021 and 30.08.2021. We aimed to reach all 35 hospitals in Croatia which have anesthesiology service available for operating theatres and/or intensive care units. Flow diagram of the study is shown in Fig 1.

The questionnaire was designed as a Google form and the survey link was distributed by e-mail (one for each hospital). If no response was received within four weeks, a reminder was sent to the hospitals. We collected answers only from anesthesiologist representatives of hospitals because in Croatia, anesthesiologists are the ones primarily responsible for advanced airway management. Only occasionally VL is used for airway management by physicians of other specialties, but it is not yet a standard practice. At the beginning of the survey, respondents were obliged to provide explicit consent for participation and publishing through a question in the questionnaire.

In our study, we used a broader definition of VL and included other rigid intubation devices that use digital or optical imaging to allow tracheal intubation, similar to previous reports [15,16]. List of VL included in our study is as follows:

- Airtraq (Prodol Meditec, Guecho, Spain)
- Bonfils endoscope (Karl Storz, Slough, UK)
- Bullard (Circon, ACMI, Stamford, CT, USA)
- C-MAC (Karl Storz, Slough, UK)
- C-MAC D-blade (Karl Storz, Slough, UK)
- GlideScope (Verathon UK, Amersham, UK)
- Infinium ClearVue (Infinium Medical, Largo, FL, USA)
- King Vision VL (Ambu, St Ives, UK)
- Levitan FPS (Clarus Medical, Minneapolis, MN, USA)
- Marshall VL (Marshall Airway Products, Radstock, UK)
- McGrath 5 (Aircraft Medical, Edinburgh, UK)
- McGrath Mac (Aircraft Medical, Edinburgh, UK)
- Pentax AWS (Pentax, Tokyo, Japan)
- Shikani stiletto (Clarus Medical, Minneapolis, MN, USA)
- Upsherscope (Mercury Medical, Clearwater, FL, USA)
- Vividtrac (Vivid Medical, Palo Alto, USA)
- Wuscope (Pentax Precision instruments, Orangeburg, NY, USA)

Participants also had options to report the use of other VL.

### Statistical analysis

Data were entered in a Microsoft Excel 2013 spreadsheet (Microsoft Corporation, Redmond, WA, USA). Statistical Package for the Social Sciences (SPSS) Statistics software, version 25.0 (IBM Corporation, Armonk, NY, USA) was used for data analysis. Data are presented as raw numbers (n) and percentages (%).

## Results

According to the Ministry of Health, there are in total 55 public hospitals in Croatia. In our study, we included all public hospitals that have anesthesiology service available. Special hospitals, like psychiatric or rehabilitation hospitals—which are primarily not involved in airway management—were excluded, as well as private institutions. The survey was eventually sent to 35 hospitals. In total, 29 duly completed forms were returned without duplicates. The response rate was 83%. The detailed characteristics of the responding hospitals are shown in Table 1.

**Table 1:** Characteristics of all the responding institutions (n = 29).

**Type of Hospital, n (%)**	Clinical Hospital Center / University Hospital County Hospital / Regional Hospital General Hospital Other	8 (28) 8 (28) 11 (38) 2 (6)
**Teaching activity of the Institution, n (%)**	Regularly (at least once per month) Occasionally (less than once per month) No involvement	18 (62) 6 (21) 5 (17)
**Availability of videolaryngoscopes, n (%)**	Yes No	25 (86) 4 (14)

Data are reported as raw numbers (n) and percentages (%).

### Availability of videolaryngoscopes

**Fig 2:**
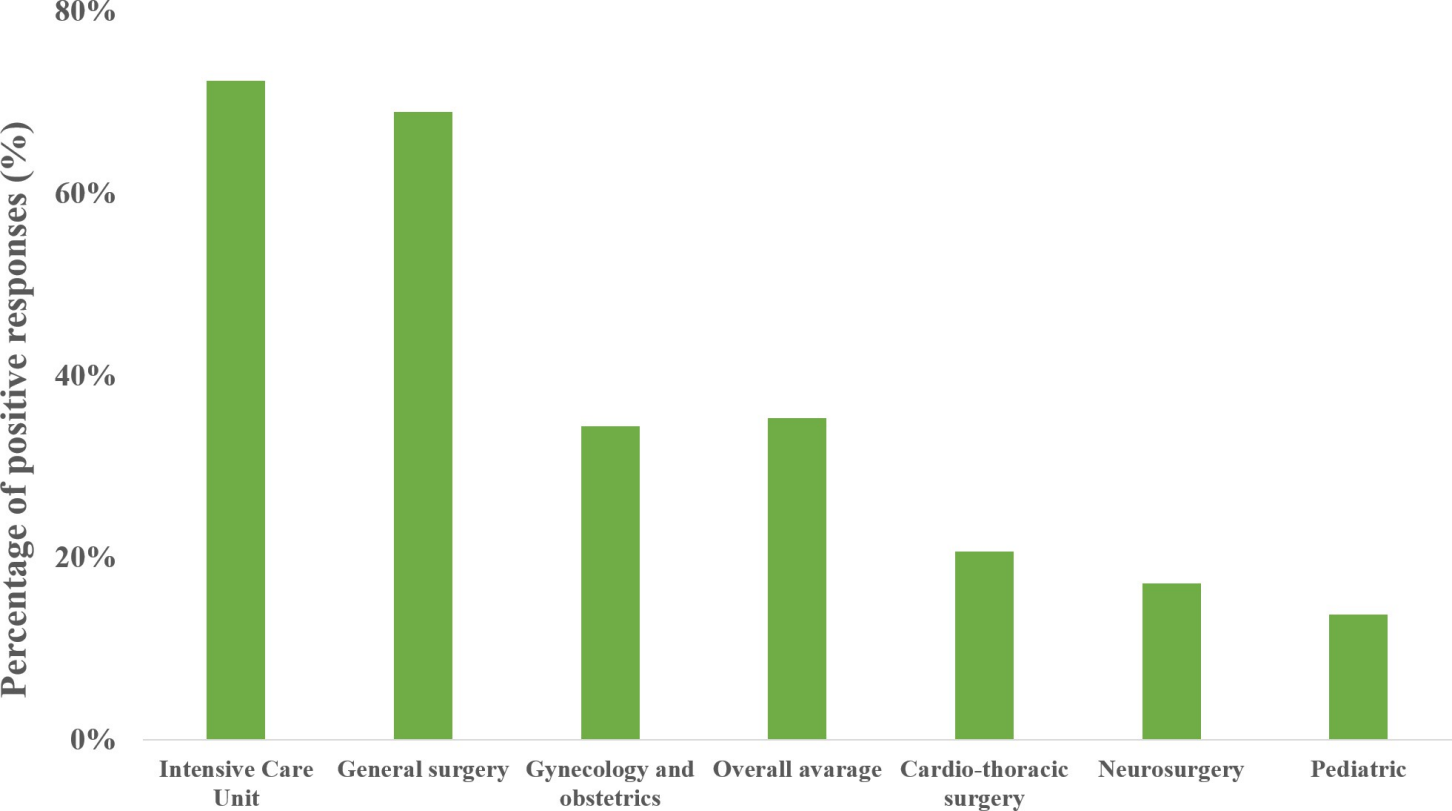
Immediate availability regarding clinical areas. The most and the least supplied clinical areas with immediate availability of VL in Croatia according to this survey (n = 29) and based on positive answers given to the following question: “At which workstation at your workplace do you have a VL immediately/readily available? (Option for multiple answers!)”.

**Fig 3:**
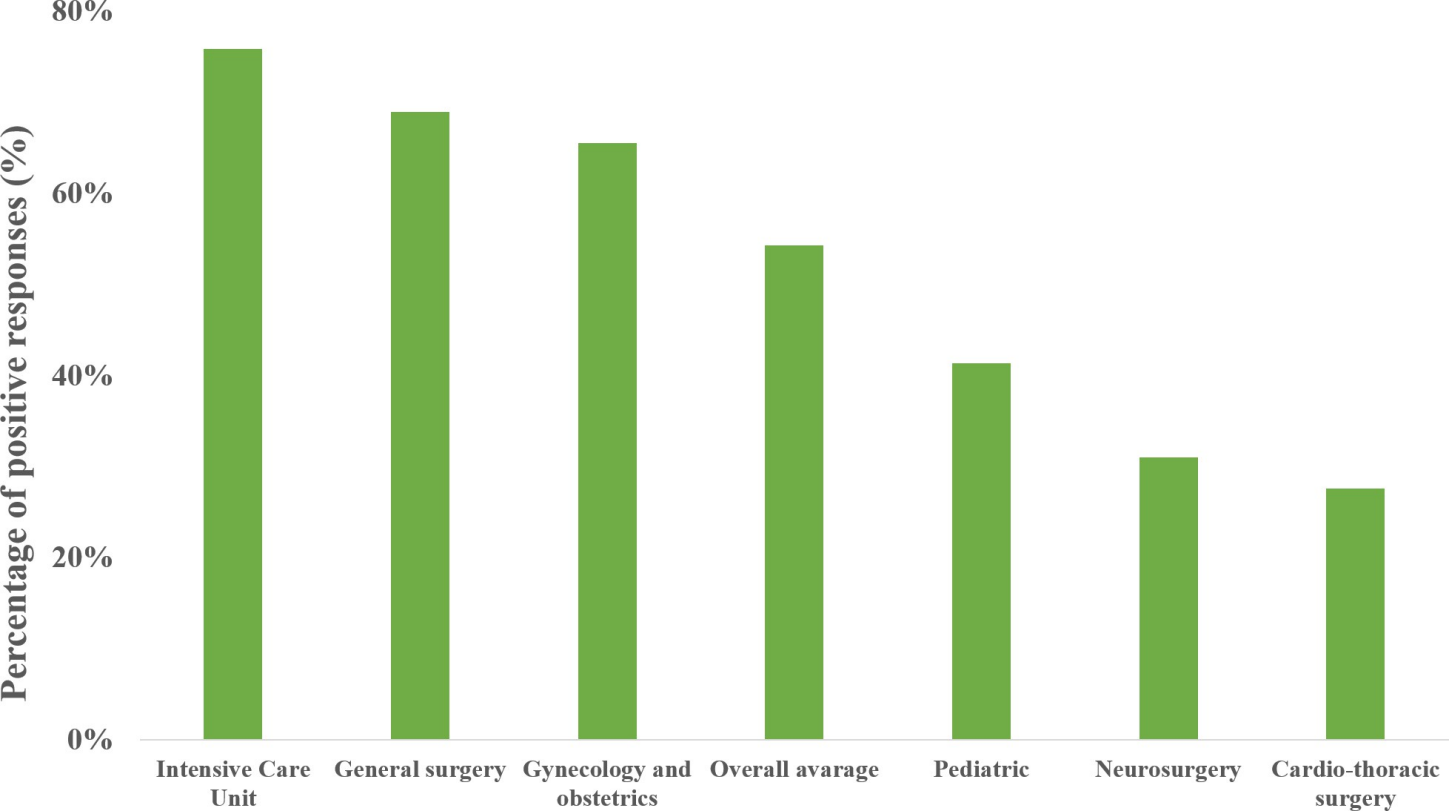
Increased time frame availability regarding clinical areas. The most and the least supplied clinical areas with availability of VL within 10 minutes in Croatia according to this survey (n = 29) and based on positive answers given to the following question: “At which workstation at your workplace do you have a VL available within 10 minutes? (Option for multiple answers!)”.

Twenty-five (86%) of the responding hospitals reported availability of VL on at least one anesthesia related clinical area (Table 1). Regarding clinical areas with immediate access to VL, reported best supplied areas were intensive care units (n = 21, 72%), general surgery (n = 20, 69%) and gynecology/obstetrics (n = 10, 34%) (Fig 2). All university hospital centers reported immediate availability of VL on at least three separate anesthesia related clinical areas. The overall average immediate availability rate was 35%. When time of availability was extended to ten minutes, the overall average availability increased to 54%. With extended time frame, order of best supplied clinical areas remained the same, while VL availability, rates were reported as follows: intensive care units (n = 21, 75%), general surgery (n = 20, 69%) and gynecology/obstetrics (n = 19, 65%) (Fig 3).

### Types and choice of videolaryngoscopes

**Fig 4:**
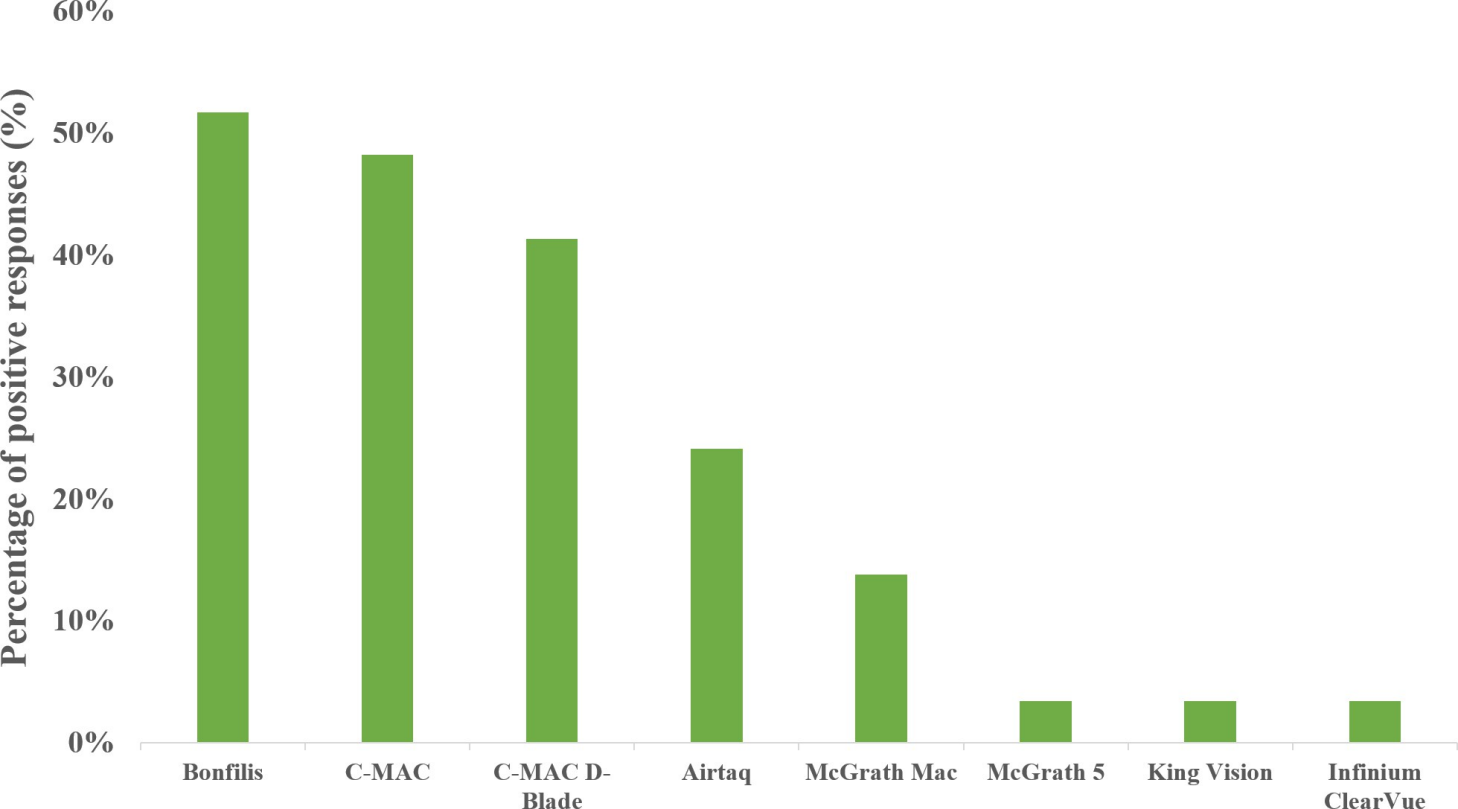
The available devices in Croatia. The eight available VL in Croatia according to this survey (n = 29) and based on positive answers given to the following question: “Which of the following devices are available at your workplace? (Option for multiple answers!)”.

Out of listed 17 devices, only 8 was named to be available in Croatian hospitals. Types of VL together with their availability are shown in Fig 4. Most available was Bonfils intubation endoscope (n = 15, 52%), while C-MAC (n = 14, 48%) and C-MAC D-Blade (n = 12, 41%) were also in three most popular devices (Fig 4).

Most common methods of choosing the device were as follows: centralized hospital procurement (n = 6, 24%), the opinion of the head of the department/institution/clinic (n = 5, 20%) and available data in the literature (n = 3, 12%). Seven of the responding hospital representatives (28%) didn’t have information on methods of choosing an available device (Table 2).

**Table 2:** Characteristics of responding institutions where videolaryngoscopes are available (n = 25).

Protocol for application of videolaryngoscopes, n (%)	Protocol available Protocol unavailable Not known	5 (20) 16 (64) 4 (16)
**Education on videolaryngoscopy, n (%)**	Mandatory education on patients Mandatory education on manakins Voluntary education on patients Introduction course by distributor Informal introduction “See one, do one, teach one” Other	2 (8) 5 (20) 4 (16) 3 (12) 7 (28) 3 (12) 1 (4)
**Choice of videolaryngoscopes, n (%)**	Centralized hospital procurement Decision of the departmental lead Based on literature Donation Previous personal experience Not known	6 (24) 5 (20) 3 (12) 2 (8) 2 (8) 7 (28)
**Applications of videolaryngoscopes, n (%)**	Routinely, in regular everyday practice In case of potentially difficult airway Backup method in case of difficult laryngoscopy For educational purposes only	4 (16) 12 (48) 8 (32) 1 (4)
**Users of videolaryngoscopes, n (%)**	Specialists in anesthesiology Residents in anesthesiology Specialists of other professions in intensive care Emergency medicine specialists Emergency medicine residents	25 (100) 20 (80) 5 (20) 2 (8) 3 (12)

Data are reported as raw numbers (n) and percentages (%).

### Training and implementation

The majority of the responding hospitals conducted some training prior introducing a specific device into clinical practice, although methods of training were diverse among institutions. Single most prevailing method of VL implementation was informal introduction (n = 7, 28%), followed by mandatory education on manikins (n = 5, 20%) (Table 2).

### Usage of videolaryngoscopy in practice

From twenty-five institutions where VL is available, only five (20%) reported to have implemented protocol for VL usage in clinical practice. 64% responded not to have any protocol, while 16% didn’t know. Only four out of twenty-five hospitals routinely use VL in everyday practice (16%), while the majority of institutions reported usage of VL occasionally, either in case of potentially difficult airway (n = 12, 48%) or as back up method following difficult direct laryngoscopy (n = 8, 32%). One hospital reported use of VL only for educational purposes. Main users of VL in responding hospitals were specialists (n = 25, 100%) and residents in anesthesiology (n = 20, 80%). Other reported specialties that use VL were as following: intensive care medicine specialists other than anesthesiologists (n = 5, 20%), emergency medicine residents (n = 3, 12%) and emergency medicine specialists (n = 2, 8%) (Table 2).

### Availability of videolaryngoscopy in COVID-19 wards

Twenty-eight of the responding hospitals are treating COVID-19 patients as well. VL was available on COVID-19 wards in 64% of hospitals (18/28), while 29% (8/28) reported not to have VL available in the COVID-19 ward. Six out of twenty-eight hospitals (21%) reported routine usage of VL in intubating COVID-19 patients. Same number of hospitals reported to use the VL occasionally for potentially difficult airway situations (n = 6, 21%), while five responded to use VL only as a backup method in case of failed direct laryngoscopy (18%) (Table 3).

**Table 3:** Characteristics of all the responding institutions where COVID-19 treatment was available (n = 28).

Videolaryngoscope availability at COVID-19 wards, n (%)	Yes No Not known	18 (64) 8 (29) 2 (7)
**Videolaryngoscope usage at COVID-19 wards, n (%)**	Not used since not available Not used even tough available Occasionally used for potentially difficult airway Occasionally used as backup Always used even for routine intubation Not known	8 (29) 1 (4) 6 (21) 5 (18) 6 (21) 2 (7)

Data are reported as raw numbers (n) and percentages (%). COVID-19: Coronavirus disease 2019.

## Discussion

Our study provides insight into the availability and use of VL in Croatian hospitals, with special consideration of COVID-19 wards. To our knowledge, this is the first investigation of that kind conducted in Croatia that included all hospitals with the response rate of 83%. The main result of our study is that VL is available in 86% of hospitals, on at least one anesthesia related clinical area. This result is in concordance with Cook et al. who found VL available in more than 90% of UK hospitals [15]. Recent study by Nagy et al. reported lower VL availability (65%) in Hungarian hospitals, but authors examined individual responses from anesthesiologists rather than hospital availability [16]. All university hospital centers, with regular teaching activities, reported to have VL immediately available in at least three separate clinical areas. Availability of VL in hospitals with regular teaching activities provides more options for optimizing airway management training of students and residents. Some data suggest VL with Macintosh-type blade could be a better teaching tool compared to direct laryngoscopes [17].

Best supplied areas are intensive care units (ICU), general surgery and gynecology/obstetrics, while the lower availability is recorded in pediatric, neurosurgery and cardio-thoracic areas. In our study, availability of VL in ICU is 72%, which is significantly higher than results from United Kingdom (54%) and Hungarian (30–44%) studies [15,16]. However, recent study showed that 76% of French ICUs have immediate access to VL, which is similar to our results, although it included only non-surgical ICUs [18]. Airway management and tracheal intubation in ICU setting are associated with a higher risk of adverse events, with a complication rate up to 45%, which can explain such high availability rate [2,19]. High VL availability in general surgery and gynecology/obstetrics areas is related to the greater incidence of difficult airway in emergency general surgery as well as in pregnant patients [20,21]. These results are in concordance with UK study. Interestingly, in a study by Nagy et al., VL availability in gynecology/obstetrics was not reported.

Most common VL devices in Croatian hospitals are Bonfils, C-MAC and C-MAC D-blade, followed by Airtraq and MacGrath Mac. Compared to other studies, Croatian results are similar to those from the United Kingdom, where C-MAC, Glidescope and Airtraq are the first three most available devices [15]. Contrary to our result where KingVision is one of the rarest devices, it was the most popular VL in Hungarian hospitals [16]. In a recent study comparing seven different VL, using devices with a Macintosh type blade (like C-MAC) scored highest in user satisfaction, as well as in time to successful intubation [22]. This may explain such popularity of these devices. Unlike in similar studies, Bonfils is the most popular alternative intubating device in Croatian hospitals. It has been in clinical practice much longer than most of VL devices and proved to be useful in difficult airway scenarios, such as unstable cervical spines, airway tumors and awake intubations [23]. It is lightweight, durable, portable and easily set up, which all are probable reasons for preference. Interestingly, all three most popular VL in Croatian hospitals are completely reusable devices, which might have economic reasons [24]. The choice of VL devices in Croatia is mainly based on centralized hospital procurement, according to overall cost, but also on the decision of the department lead. Contrary to our results, central hospital procurement wasn’t reported in previous studies as main approach of choosing VL.

Methods of introducing and training of VL varied in our research, informal introduction being the most prevalent method. French Society of Anaesthesia and Intensive Care Medicine (SFAR) recommend initial theoretical introduction followed by practice on manikins and simulators until achieving proficiency [25]. Although VL as a skill is easily learned, performance outcome is influenced by the quality of training, access to the device and deliberate practice to achieve expertise [26]. Even though necessary, skill assessment for VL is difficult because competencies for various devices are still not well defined [27,28].

Recent Cochrane systematic review concluded that VL improves the glottic view, reduces number of intubation attempts and may reduce airway trauma as well [29]. Although VL advantages are recognized, it is still not clear whether this would result in fewer incidents of hypoxemia or respiratory complications. Use of VL in Croatian hospitals is mostly occasional, in cases of potentially difficult airway or as a backup method after failed intubation. Main users are specialists and residents in anesthesiology, but VL is also used in few institutions by intensivists and emergency medicine specialists. Only 16% of hospitals reported regular use in everyday anesthesia practice. Similarly, UK study showed that 50% of those who have VL available for anesthesia reported infrequent use and none reported universal use [15]. Reasons for this may be inhomogeneous availability across clinical areas, varying in devices, lack of protocols and lack of structured and continuous training.

Due to enhanced visualization of upper airway anatomical structures, use of VL expanded beyond airway management. VL was successfully used in surgical treatment of epiglottic cysts, vocal cord biopsies and tongue base surgery [30–32]. Published data suggest that VL might shorten duration of these surgical procedures, while minimizing adverse events occurence. Furthermore, VL has been successfully used to remove foreign bodies from hypopharynx, larynx and upper esophagus, both in adult and pediatric patients [33–35].

The COVID-19 pandemic, during last two years, caused a surge of critically ill patients requiring emergency tracheal intubation, additionally challenged by remote-site working, working under personal protection equipment (PPE) and health risk of viral exposure. Due to certain advantages, including the limiting risk of infection spread, improved visibility under PPE and lower risk of failed intubation, VL became recommended method of endotracheal intubation in COVID-19 patients [10,11,13]. In our study, 64% of hospitals reported to have VL available in COVID-19 wards. Despite current guidelines that encourage using VL, only 21% of Croatian hospitals use VL always, in routine intubation of COVID-19 patients. Compared to our results, Cohen et al. reported that VL was the default device used in intubation of COVID-19 patients in 93% of anesthesia departments in Israel [36]. Single-center study from the UK found that 79% of all intubations of COVID-19 patients were done with VL [37]. The reasons for our low availability of VL and low rate of routine usage probably lie in relying on the more familiar skill of direct laryngoscopy in the setting of displaced COVID-19 wards, but warrants further research.

Limitation of our study may be that the survey was addressed to the hospital representatives (head of department or senior anesthesiologist) and not to each anesthesiologist, who are the link between the patients and the available devices, although UK research had a similar protocol to ours. However, the individual approach of the Hungarian study showed the low response rate (21%), compared to UK research (67%).

## Conclusion

Despite numerous VL devices are available in the market, only eight are in use in Croatia. Our study also showed that VL is available in the majority of Croatian hospitals, but VL use is still mainly restricted to difficult airway scenarios. Use of VL in COVID-19 management in Croatia is also low and education on the method is still mainly informal. Based upon our results better implementation in practice should be targeted, as well as formal skill trainings especially regarding COVID-19 care.

## Supporting information

S1 Table Videolaryngoscopy dataset. (XLSX)

S1 Appendix Survey Croatian version. (DOCX)

S2 Appendix Survey English version. (DOCX)

## References

[1] Schroeder RA, Pollard R, Dhakal I, Cooter M, Aronson S, Grichnik K, et al. Temporal trends in difficult and failed tracheal intubation in a regional community anesthetic practice. Anesthesiology. 2018. doi: 10.1097/ALN.0000000000001974 29189209

[2] Cook TM, Woodall N, Frerk C. Major Complications of Airway management in the United Kingdom. Report and Findings. Fourth National Audit Project of the Royal College of Anaesthetists and Difficult Airway Society. 2011. doi: 10.1093/bja/aer058 21447488

[3] Garcia-Marcinkiewicz AG, Kovatsis PG, Hunyady AI, Olomu PN, Zhang B, Sathyamoorthy M, et al. First-attempt success rate of video laryngoscopy in small infants (VISI): a multicentre, randomised controlled trial. Lancet. 2020. doi: 10.1016/S0140-6736(20)32532-0 33308472

[4] Aziz MF, Brambrink AM, Healy DW, Willett AW, Shanks A, Tremper T, et al. Success of Intubation Rescue Techniques after Failed Direct Laryngoscopy in Adults. Anesthesiology. 2016. doi: 10.1097/ALN.0000000000001267 27483124

[5] Zaouter C, Calderon J, Hemmerling TM. Videolaryngoscopy as a new standard of care. British Journal of Anaesthesia. 2015. doi: 10.1093/bja/aeu266 25150988

[6] Buis ML, Maissan IM, Hoeks SE, Klimek M, Stolker RJ. Defining the learning curve for endotracheal intubation using direct laryngoscopy: A systematic review. Resuscitation. 2016. doi: 10.1016/j.resuscitation.2015.11.005 26711127

[7] Cordovani D, Russell T, Wee W, Suen A, Cooper RM. Measurement of forces applied using a Macintosh direct laryngoscope compared with a Glidescope video laryngoscope in patients with predictors of difficult laryngoscopy: A randomised controlled trial. Eur J Anaesthesiol. 2019. doi: 10.1097/EJA.0000000000000901 30308524

[8] Luo M, Cao S, Wei L, Tang R, Hong S, Liu R, et al. Precautions for Intubating Patients with COVID-19. Anesthesiology. 2020. doi: 10.1097/ALN.0000000000003288 32195703 PMC7155910

[9] Brown J, Gregson FKA, Shrimpton A, Cook TM, Bzdek BR, Reid JP, et al. A quantitative evaluation of aerosol generation during tracheal intubation and extubation. Anaesthesia. 2021. doi: 10.1111/anae.15292 33022093 PMC7675579

[10] Alhazzani W, Møller MH, Arabi YM, Loeb M, Gong MN, Fan E, et al. Surviving Sepsis Campaign: Guidelines on the Management of Critically Ill Adults with Coronavirus Disease 2019 (COVID-19). Crit Care Med. 2020. doi: 10.1097/CCM.0000000000004363 32224769 PMC7176264

[11] Wax RS, Christian MD. Practical recommendations for critical care and anesthesiology teams caring for novel coronavirus (2019-nCoV) patients. Canadian Journal of Anesthesia. 2020. doi: 10.1007/s12630-020-01591-x 32052373 PMC7091420

[12] Frerk C, Mitchell VS, McNarry AF, Mendonca C, Bhagrath R, Patel A, et al. Difficult Airway Society 2015 guidelines for management of unanticipated difficult intubation in adults. Br J Anaesth. 2015. doi: 10.1093/bja/aev371 26556848 PMC4650961

[13] Foley LJ, Urdaneta F, Berkow L, Aziz MF, Baker PA, Jagannathan N, et al. Difficult Airway Management in Adult Coronavirus Disease 2019 Patients: Statement by the Society of Airway Management. Anesth Analg. 2021. doi: 10.1213/ANE.0000000000005554 33711004

[14] Cook TM, El-Boghdadly K, McGuire B, McNarry AF, Patel A, Higgs A. Consensus guidelines for managing the airway in patients with COVID-19: Guidelines from the Difficult Airway Society, the Association of Anaesthetists the Intensive Care Society, the Faculty of Intensive Care Medicine and the Royal College of Anaesthetist. Anaesthesia. 2020. doi: 10.1111/anae.15054 32221970 PMC7383579

[15] Cook TM, Kelly FE. A national survey of videolaryngoscopy in the United Kingdom. Br J Anaesth. 2017. doi: 10.1093/bja/aex052 28403414

[16] Nagy B, Rendeki S. A national survey of videolaryngoscopes and alternative intubation devices in Hungary. PLoS One. 2019. doi: 10.1371/journal.pone.0223645 31600304 PMC6786552

[17] Kelly FE, Cook TM. Seeing is believing: Getting the best out of videolaryngoscopy. British Journal of Anaesthesia. 2016. doi: 10.1093/bja/aew052 27095240

[18] Martin M, Decamps P, Seguin A, Garret C, Crosby L, Zambon O, et al. Nationwide survey on training and device utilization during tracheal intubation in French intensive care units. Ann Intensive Care. 2020. doi: 10.1186/s13613-019-0621-9 31900637 PMC6942097

[19] Russotto V, Myatra SN, Laffey JG, Tassistro E, Antolini L, Bauer P, et al. Intubation Practices and Adverse Peri-intubation Events in Critically Ill Patients from 29 Countries. JAMA—J Am Med Assoc. 2021. doi: 10.1001/jama.2021.1727 33755076 PMC7988368

[20] Howle R, Onwochei D, Harrison SL, Desai N. Comparison of videolaryngoscopy and direct laryngoscopy for tracheal intubation in obstetrics: a mixed-methods systematic review and meta-analysis. Canadian Journal of Anesthesia. 2021. doi: 10.1007/s12630-020-01908-w 33438172

[21] Schnittker R, Marshall SD, Berecki-Gisolf J. Patient and surgery factors associated with the incidence of failed and difficult intubation. Anaesthesia. 2020. doi: 10.1111/anae.14997 32232991

[22] Pieters BMA, Wilbers NER, Huijzer M, Winkens B, Van Zundert AAJ. Comparison of seven videolaryngoscopes with the Macintosh laryngoscope in manikins by experienced and novice personnel. Anaesthesia. 2016. doi: 10.1111/anae.13413 26973253

[23] Thong SY, Wong TGL. Clinical uses of the Bonfils Retromolar Intubation Fiberscope: a review. Anesth Analg. 2012;115: 855–66. doi: 10.1213/ANE.0b013e318265bae2 22956530

[24] McGain F, Story D, Lim T, McAlister S. Financial and environmental costs of reusable and single-use anaesthetic equipment. Br J Anaesth. 2017. doi: 10.1093/bja/aex098 28505289

[25] Quintard H, l’Her E, Pottecher J, Adnet F, Constantin JM, De Jong A, et al. Experts’ guidelines of intubation and extubation of the ICU patient of French Society of Anaesthesia and Intensive Care Medicine (SFAR) and French-speaking Intensive Care Society (SRLF): In collaboration with the pediatric Association of French-speaking A. Ann Intensive Care. 2019. doi: 10.1186/s13613-019-0483-1 30671726 PMC6342741

[26] Armstrong L, Harding F, Critchley J, McNarry AF, Myatra SN, Cooper R, et al. An international survey of airway management education in 61 countries†. Br J Anaesth. 2020. doi: 10.1016/j.bja.2020.04.051 32444066

[27] Landucci F, Caldiroli D, Chiumello D, Byrne A. Cognitive resistance towards videolaryngoscopy⋯ and why macintosh refuses to die. Minerva Anestesiologica. 2017. doi: 10.23736/S0375-9393.17.12211-X 28631464

[28] Behringer EC, Cooper RM, Luney S, Osborn IP. The comparative study of video laryngoscopes to the Macintosh laryngoscope: Defining proficiency is critical. European Journal of Anaesthesiology. 2012. doi: 10.1097/EJA.0b013e32834c46c8 21968635

[29] Lewis SR, Butler AR, Parker J, Cook TM, Schofield-Robinson OJ, Smith AF. Videolaryngoscopy versus direct laryngoscopy for adult patients requiring tracheal intubation: A Cochrane Systematic Review. British Journal of Anaesthesia. 2017. doi: 10.1093/bja/aex228 28969318

[30] Meng X, Wen Q, Gu J, Wang Y. Videolaryngoscope-assisted coblation of epiglottic cysts. Eur Arch Oto-Rhino-Laryngology. 2020. doi: 10.1007/s00405-020-05804-3 31993766

[31] Bruno E, Dauri M, Mauramati S, Viziano A, Micarelli A, Ottaviani F, et al. Utility of glidescope® videolaryngoscopy in surgical procedures involving the larynx. Acta Otorhinolaryngol Ital. 2015 Feb; 35(1): 45–48. 26015651 PMC4443577

[32] Shenoy PK, Aldea M. The use of GlideScope for biopsies of the tongue base. J Laryngol Otol. 2013. doi: 10.1017/S002221511200271X 23217214

[33] Cagini L, Ragusa M, Vannucci J, Andolfi M, Cirulli P, Scialpi M, et al. Glide video laryngoscope for the management of foreign bodies impacted at the hypopharyngeal level in adults. Minerva Anestesiol. 2013 Nov;79(11):1259–63. 23811626

[34] Kaufmann J, Grozeva B, Laschat M, Brackhahn M, Rudolph D, Neuhaus N, et al. Rapid and safe removal of foreign bodies in the upper esophagus in children using an optimized Miller size 3 video laryngoscope blade. Paediatr Anaesth. 2021. doi: 10.1111/pan.14158 33583069

[35] Ho G, Singh N, Andrews J, Westhead P. Novel use of videolaryngoscopy to remove a foreign body. BMJ Case Rep. 2015. doi: 10.1136/bcr-2015-210011 26153286 PMC4499719

[36] Cohen B, Baar Y, Fein S, Matot I. Anesthesia departments’ readiness for the COVID-19 pandemic; a nationwide cross-sectional study in Israel. BMC Anesthesiol. 2020. doi: 10.1186/s12871-020-01173-w 33050885 PMC7552575

[37] Gandhi A, Sokhi J, Lockie C, Ward PA. Emergency Tracheal Intubation in Patients with COVID-19: Experience from a UK Centre. Anesthesiol Res Pract. 2020. doi: 10.1155/2020/8816729 33376486 PMC7729388

