# Supplementary material for: Croatian national audit on videolaryngoscopes and alternative intubation devices in the era of COVID-19 pandemic
Source: PLoS One. 2023 Jan 11;18(1):e0280236. doi: 10.1371/journal.pone.0280236 (PMC9833575; doi:10.1371/journal.pone.0280236)
Supplement: S1 Appendix — (DOCX) [file pone.0280236.s002.docx]

**Anketni upitnik**

Poštovani kolegice/kolega,

Zahvaljujemo na odvojenom vremenu i Vašem sudjelovanju u ovom anketnom istraživanju. Videolaringoskopija postaje sve raširenija metoda osiguranja dišnog puta u posljednjih desetak godina. Istraživanja su pokazala njene prednosti kako u situacijama otežanog dišnog puta tako i u rutinskoj primjeni u pojedinih specifičnih skupina bolesnika. Ovo istraživanje ima za cilj utvrditi dostupnost ove tehnologije anesteziolozima u hrvatskim bolnicama.

Anketni upitnik ispituje dostupnost videolaringoskopije u Vašoj bolnici ili odjelu, kao i način uvođenja same metode u Vašu ustanovu i provedene edukacije. Potrebno je otprilike 10 minuta Vašeg vremena za popunjavanje upitnika. Ispunjavanjem upitnika pristajete na sudjelovanje u ovom anketnom istraživanju te na objavljivanje rezultata u znanstvenim časopisima.

**Kojoj skupini pripada Vaša bolnica?**

Klinički bolnički centar / Sveučilišna bolnica

Županijska bolnica / Regionalna bolnica

Opća bolnica

Isključivo dječja bolnica

Isključivo bolnica za ginekologiju i porodništvo

Drugo (molim navesti vrstu ustanove):

**Da li se u Vašoj bolnici provodi organizirana edukacija specijalizanata ili studenata?**

Edukacija se provodi redovno

Edukacija se provodi povremeno ( rjeđe od jednom mjesečno)

Bolnica nije uključena u edukaciju studenata ni specijalizanata

**Da li u Vašoj bolnici postoji bilo koja vrsta videolaringoskopskog uređaja, bez obzira na kojem radilištu?**

Da

Ne

Ne znam

**Na kojem je od navedenih radilišta u Vašoj bolnici odmah dostupan videolaringoskop? (moguće je više odgovora)**

Na nijednom radilištu

Opća kirurgija

Ginekologija i porodništvo

Otorinolaringologija / Maksilofacijalna kirurgija

Dječja kirurgija

Jedinica intenzivnog liječenja

Hitni prijem

Kardiokirurgija / Torakalna kirurgija

Neurokirurgija

Drugo ( molim navesti):

**Na kojem od navedenih radilišta u Vašoj bolnici videolaringoskop može biti dostupan unutar 10 minuta? (moguće je više odgovora)**

Na nijednom radilištu

Opća kirurgija

Ginekologija i porodništvo

Otorinolaringologija / Maksilofacijalna kirurgija

Dječja kirurgija

Jedinica intenzivnog liječenja

Hitni prijem

Kardiokirurgija / Torakalna kirurgija

Neurokirurgija

Drugo ( molim navesti):

**Koji od navedenih videolaringoskopa je dostupan u Vašoj bolnici ? (moguće je više odgovora)**

Nema dostupnog videolaringoskopa u bolnici

Postoji videolaringoskop, ali nisam siguran/na o kojem uređaju se radi

Airtraq (Prodol Meditec, Guecho, Spain)

Bonfils stilet(Karl Storz, Slough, UK)

Bullard (Circon, ACMI, Stamford, CT, USA)

C-MAC (Karl Storz, Slough, UK)

C-MAC D-blade (Karl Storz, Slough, UK)

GlideScope (Verathon UK, Amersham, UK)

Infinium ClearVue (Infinium Medical, Largo, FL, USA)

King Vision VL (Ambu, St Ives, UK)

Levitan FPS (Clarus Medical, Minneapolis, MN, USA)

Marshall VL ( Marshall Airway Products, Radstock, UK)

McGrath 5 (Aircraft Medical, Edinburgh, UK)

McGrath Mac (Aircraft Medical, Edinburgh, UK)

Pentax AWS (Pentax, Tokyo, Japan)

Shikani stilet (Clarus Medical, Minneapolis, MN, USA)

Upsherscope (Mercury Medical, Clearwater, FL, USA)

Vividtrac (Vivid Medical, Palo Alto, USA)

Wuscope (Pentax Precision instruments, Orangeburg, NY, USA)

Drugi (molim navesti koji):

**Kako je odabran videolaringoskop koji je dostupan u Vašoj bolnici?**

Ne postoji dostupan videolaringoskop u bolnici

Temeljem cijene

Temeljem dostupnih podataka u stručnoj literaturi

Temeljem provedenog kliničkog ispitivanja u Vašoj bolnici

Temeljem mišljenja lokalnih stručnjaka za dišni put

Temeljem mišljenja voditelja odjela/zavoda/klinike

Temeljem centralizirane bolničke nabave

Donacijom izvan bolničkog sustava

Na neki drugi način (molim navesti koji):

Ne znam

**Na koji način je provedena edukacija o korištenju videolaringoskopa koji postoji u Vašoj bolnici?**

Nije dostupan videolaringoskop

Nije postojala nikakva formalna edukacija u bolnici

Po principu „pogledaj jednom, učini jednom, poučavaj jednom“ ( eng. „see one, do one, teach one“)

Neformalno upoznavanje s radom uređaja

Obavezna edukacija na lutkama

Dobrovoljna edukacija na lutkama

Obavezna edukacija na pacijentima

Dobrovoljna edukacija na pacijentima

Drugo (molim navesti koje):

**U kojim slučajevima se, po Vašoj procjeni, koriste videolaringoskopi u Vašoj bolnici?**

Rutinski, u redovnom radu

Povremeno, u slučajevima potencijalno otežanog dišnog puta

Povremeno, povremeno kao rezervna metoda u slučaju otežane direktne laringoskopije

U edukacijske svrhe

Ne koristi se, iako je dostupan

Ne koristi se i nije dostupan

**Po Vašim saznanjima, koji liječnici u Vašoj bolnici koriste videolaringoskopiju? (moguće je više odgovora)**

Nije dostupan videolaringoskop

Specijalisti anesteziologije, reanimatologije i intenzivne medicine

Specijalizanti anesteziologije, reanimatologije i intenzivne medicine

Specijalisti drugih struka u jedinicama intenzivnog liječenja – intenzivisti

Specijalizanti drugih struka u jedinicama intenzivnog liječenja

Specijalisti hitne medicine

Specijalizanti hitne medicine

Drugo (molim navesti):

**Da li Vaša bolnica ima protokol korištenja videolaringoskopa?**

Da

Ne

Ne znam

**Da li Vaša bolnica ima dostupan videolaringoskop u odjelima za COVID-19 bolesnike?**

Da

Ne

Ne znam

U bolnici se ne liječe COVID-19 bolesnici

**Da li se u Vašoj bolnici videolaringoskop koristi za intubaciju COVID-19 bolesnika?**

Da, rutinski za svaku takvu intubaciju

Da, povremeno u slučaju potencijalno otežane intubacije

Da, povremeno kao rezervna metoda u slučaju otežane direktne laringoskopije

Ne koristi se, iako je dostupan

Ne koristi se i nije dostupan

Ne znam
