# Supplementary material for: Croatian national audit on videolaryngoscopes and alternative intubation devices in the era of COVID-19 pandemic
Source: PLoS One. 2023 Jan 11;18(1):e0280236. doi: 10.1371/journal.pone.0280236 (PMC9833575; doi:10.1371/journal.pone.0280236)
Supplement: S2 Appendix — (DOCX) [file pone.0280236.s003.docx]

**Survey**

Dear Colleague,

Thank You for taking time to participate in this research.

In the last ten years, videolaryngoscopy is becoming increasingly popular airway management method. Based on recent studies, it offers some benefit in difficult airway situations, as well as in routine use in specific patient subgroups. This research aims to investigate data on videolaryngoscopy availability to anesthesiologists in Croatian hospitals. Questions in this survey will establish availability of videolaryngoscopy in Your hospital and/or department, as well as the method of device implementation into practice and skill training. Estimated time to complete the survey is ten minutes. By answering the survey questions, You consent to participate in this research and for data to be published.

**Which group does your hospital belong to?**

Clinical Hospital Center / University Hospital

County Hospital / Regional Hospital

General Hospital

Exclusively children's hospital

Exclusively hospital for gynecology and obstetrics

Other (please specify type of institution):

**Is there an organized education of residents or students in your hospital?**

Education is conducted regularly

Education is conducted occasionally (less than once a month)

The hospital is not involved in the education of students or residents

**Is there any type of videolaryngoscopic device in your hospital, regardless of which worksite?**

Yes

No

I do not know

**At which of the following worksites in your hospital is a videolaryngoscope immediately available? (multiple answer possible)**

Nowhere

General surgery

Gynecology and obstetrics

Otorhinolaryngology / Maxillofacial surgery

Pediatric surgery

Intensive care unit

Emergency department

Cardiac surgery / Thoracic surgery

Neurosurgery

Other (please specify):

**At which of the following worksites in your hospital can a videolaryngoscope be available within 10 minutes? (multiple answer possible)**

Nowhere

General surgery

Gynecology and obstetrics

Otorhinolaryngology / Maxillofacial surgery

Pediatric surgery

Intensive care unit

Emergency department

Cardiac surgery / Thoracic surgery

Neurosurgery

Other (please specify):

**Which of the following videolaryngoscopes is available at your hospital? (multiple answer possible)**

No videolaryngoscope is available at the hospital

There is a videolaryngoscope, but I'm not sure which device it is

Airtraq (Prodol Meditec, Guecho, Spain)

Bonfils stiletto (Karl Storz, Slough, UK)

Bullard (Circon, ACMI, Stamford, CT, USA)

C-MAC (Karl Storz, Slough, UK)

C-MAC D-blade (Karl Storz, Slough, UK)

GlideScope (Verathon UK, Amersham, UK)

Infinium ClearVue (Infinium Medical, Largo, FL, USA)

King Vision VL (Ambu, St Ives, UK)

Levitan FPS (Clarus Medical, Minneapolis, MN, USA)

Marshall VL (Marshall Airway Products, Radstock, UK)

McGrath 5 (Aircraft Medical, Edinburgh, UK)

McGrath Mac (Aircraft Medical, Edinburgh, UK)

Pentax AWS (Pentax, Tokyo, Japan)

Shikani stiletto (Clarus Medical, Minneapolis, MN, USA)

Upsherscope (Mercury Medical, Clearwater, FL, USA)

Vividtrac (Vivid Medical, Palo Alto, USA)

Wuscope (Pentax Precision instruments, Orangeburg, NY, USA)

Others (please specify which):

**How is the videolaryngoscope available at your hospital selected?**

There is no videolaryngoscope available at the hospital

Based on price

Based on available data in the literature

Based on a clinical trial conducted at your hospital

Based on the opinions of local airway experts

Based on the opinion of the head of the department / institution / clinic

Based on centralized hospital procurement

By donation outside the hospital system

In another way (please specify which):

I do not know

**How was the education on the use of the videolaryngoscope that exists in your hospital conducted?**

No videolaryngoscope is available at the hospital

There was no formal education at the hospital

According to the principle "see one, do one, teach one"

Informal introduction to the operation of the device

Mandatory education on manakins

Voluntary education on manakins

Mandatory education on patients

Voluntary education on patients

Other (please specify which):

**In which cases do you think videolaryngoscopes are used in your hospital?**

Routinely, in regular everyday practice

Occasionally, in cases of potentially difficult airway

Occasionally, as a backup method in case of difficult direct laryngoscopy

For educational purposes

Not used, although available

Not used and not available

**To your knowledge, which doctors in your hospital use videolaryngoscopy? (multiple answer possible)**

No videolaryngoscope is available at the hospital

Specialists in anesthesiology, resuscitation and intensive care

Residents in anesthesiology, resuscitation and intensive care

Specialists of other professions in intensive care units - intensivists

Residents of other professions in intensive care units

Emergency medicine specialists

Emergency medicine residents

Other (please specify):

**Does your hospital have a videolaryngoscope use protocol?**

Yes

No

I do not know

**Does your hospital have a videolaryngoscope available in the wards for COVID-19 patients?**

Yes

No

I do not know

COVID-19 patients are not treated in the hospital

**Is videolaryngoscope used in your hospital to intubate COVID-19 patients?**

Yes, routinely for any such intubation

Yes, occasionally in case of potentially difficult intubation

Yes, occasionally as a backup method in case of difficult direct laryngoscopy

Not used, although available

Not used and not available

I do not know
